# Incidence and severity of nonionic low-osmolar iodinated contrast medium-related adverse drug reactions in the Republic of Korea: Comparison by generic

**DOI:** 10.1097/MD.0000000000033717

**Published:** 2023-05-12

**Authors:** Eun Bee Jang, Chong Hyun Suh, Pyeong Hwa Kim, Ah Young Kim, Kyung-Hyun Do, Jeong Hyun Lee, Dong-Il Gwon, Ah Young Jung, Choong Wook Lee

**Affiliations:** a Department of Radiology and Research Institute of Radiology, University of Ulsan College of Medicine, Asan Medical Center, Seoul, Republic of Korea.

**Keywords:** contrast media, drug-related side effects and adverse reaction, republic of Korea, tomography, X-ray computed

## Abstract

We aimed to report the incidence and severity of nonionic low-osmolar iodine contrast medium (ICM)-related adverse drug reactions (ADRs) in the Republic of Korea, by analyzing data from our single tertiary institution and published Korean reports, and to determine whether there is a difference in the incidence of ICM-related ADR by ICM generics. A total of 1,161,419 consecutive contrast-enhanced computed tomography (CT) examinations between January 2016 and December 2021 at Asan Medical Center were included. A systematic search of the literature investigating the incidence of ICM-related ADR in the Republic of Korea published up to December 31, 2021 was performed. We pooled these outcomes with those of our study using a binomial-normal model, and the pooled incidences of ADRs were compared among ICM generics using chi-square tests. Seven studies with a total of 2,570,986 contrast-enhanced CT examinations from 12 institutions were included. The pooled incidences of overall, mild, moderate, and severe ICM-related ADRs in the Republic of Korea were 0.82% (95% CI: 0.61%–1.10%), 0.72% (95% CI: 0.50%–1.04%), 0.11% (95% CI: 0.08%–0.15%), and 0.013% (95% CI: 0.010%–0.018%), respectively. In multiple pairwise comparisons, there were no significant differences in the overall incidence of ADRs between ICM generics, except iomeprol versus iobitridol and iomeprol versus iohexol. For moderate and severe ADRs, there were no significant differences in ADR incidence between ICM generics. The incidence of moderate and severe ICM-related ADRs did not differ among ICM generics. Our results suggest that no restriction is required for selection among nonionic low-osmolar ICMs.

## 1. Introduction

Contrast-enhanced computed tomography (CT) is increasingly used because contrast agents can improve the soft tissue contrast, thereby increasing diagnostic accuracy.^[1]^ With the development of nonionic low-osmolar iodinated contrast media (ICM), the incidence of ICM-related adverse drug reactions (ADRs) has decreased.^[2,3]^ For example, in previous study, the incidence of ADRs was 6% to 8% for ionic agents (used between 1985 and 1986) and 0.2% for nonionic agents (used between 1991 and 1999).^[3]^ However, given the increase in ICM use, the total number of ICM-related ADRs, the leading form of drug-related ADRs, remains important.^[1,4]^ Most ICM-related ADRs involve mild symptoms, such as urticaria, nausea, and vomiting.^[5]^ However, severe ADRs, including anaphylaxis, can be life-threatening.^[6,7]^ Since it is difficult to predict ICM-related ADR occurrence,^[8]^ systematic prevention and management of ICM-related ADRs, by identifying their incidence and associated factors, are essential.^[9]^

Previous studies have reported the incidence of ICM-related ADRs as 0.16% to 7.99% for nonionic low-osmolar ICMs.^[10]^ In studies of patient-related risk factors, a prior ICM-related ADR was identified as the greatest risk factor for future ICM-related ADR.^[11,12]^ Factors related to ICM itself have also been studied; however, the reported incidences of ADRs for each ICM generic were inconsistent across studies. For instance, Koh et al reported that the incidences of ADRs differed significantly among ICMs,^[7]^ whereas Lee et al found no significant difference.^[13]^

Recently, HLA-DRB1*15:02 was identified as a genetic risk factor for ICM-related anaphylaxis in the Korean population, demonstrating the potential for genetic susceptibility in ICM-related ADRs.^[14]^ Therefore, as a single ethnic group, it is necessary to organize ICM-related ADRs separately for the population of the Republic of Korea. We investigated the incidence and severity of ICM-related ADRs in the Republic of Korea by analyzing data from Asan Medical Center and published studies based on Korean population, and evaluated whether there is a difference in the incidence of ICM-related ADRs for different ICM generics.

## 2. Materials and methods

This retrospective study was approved by the Institutional Review Board of Asan Medical Center, and the requirement for informed consent was waived.

### 2.1. Data collection

Consecutive contrast-enhanced CT examinations using nonionic low-osmolar ICMs between January 2016 and December 2021 at a single tertiary institution were identified. The study period was determined based on the time when the problem reporting system was established in electronic medical record at our institution. Those performed in pediatric patients and in patients during hospitalization in the general ward, intensive care unit, or emergency room were excluded. Because it was hypothesized that the high proportion of severe cases among hospitalized patients in this institution may cause selection bias, only outpatient CT examinations were included. All CT examinations included electronic medical records of the brand and generic profile of the ICM used, administration of premedication, the presence of ICM-related ADRs, and, if any, the symptoms and management of the ADR.

### 2.2. ICM policy in Asan medical center

Six nonionic low-osmolar ICMs were used intravenously for CT during the study period: iobitridol (Xenetix, Guerbet, Aulnay, France), iohexol (Bonorex, Central Medical Service, Seoul, Republic of Korea; Omnipaque, GE Healthcare, Milwaukee, WI), iomeprol (Iomeron, Bracco, Milan, Italy), iopamidol (Iopamiro, Bracco, Milan, Italy; Pamiray, Dongkook Pharmaceutical, Seoul, Republic of Korea), iopromide (Ultravist, Bayer Healthcare, Berlin, Germany), and ioversol (Optiray, Mallinck-rodt Medical, St Louis, MO). Ioversol has been used since June 2017. As a result of competitive bidding, iobitridol, iomeprol, and iopromide were discontinued as first-line agents during the study period and were used as second-line agents when contrast medium replacement was required due to ADR to first-line agents. To ensure unbiased measures, data for iobitridol, iomeprol, and iopromide were excluded. For each CT protocol, the type of contrast agent, the injection rate, the dose per kilogram, and the iodine concentration were fixed, and even after changing the contrast agent, other details remained the same. Even in the cases that patients had experienced only mild symptoms, such as local urticaria, after a previous examination, premedication was performed prior to re-exposure in all cases, unless the patient refused the premedication. Contrast medium replacement was considered if moderate-to-severe ICM-related ADR had occurred previously.

### 2.3. ICM-related ADRs

The definition and severity classification of ICM-related ADR was based on the American College of Radiology manual.^[15]^ The signs and symptoms of ADR were classified as follows: mild, self-limited signs and symptoms without progression (e.g., limited urticaria/pruritis, cutaneous edema, limited itchy/scratchy throat, nasal congestion, sneezing/conjunctivitis/rhinorrhea, limited nausea/vomiting, transient flushing/warmth/chills, and headache/dizziness); moderate, more pronounced signs and symptoms requiring medical management (e.g., diffuse urticaria/pruritis, diffuse erythema with stable vital signs, facial edema without dyspnea, throat tightness or hoarseness without dyspnea, wheezing without hypoxia, protracted nausea/vomiting, and isolated chest pain); severe, life-threatening signs and symptoms that can result in permanent morbidity if not managed appropriately (e.g., diffuse edema with dyspnea, diffuse erythema with hypotension, laryngeal edema with stridor/hypoxia, wheezing with hypoxia, anaphylactic shock, arrhythmia, and convulsion). Immediate and delayed adverse reactions were pooled and analyzed together, without isolation. Physiologic reactions and allergic-like reactions were not distinguished because they overlapped and it was difficult to distinguish them in clinical practice.

### 2.4. Study design and statistical analyses

#### 2.4.1. Incidence of ICM-related ADR in Asan medical center

The incidence of ICM-related ADR was calculated for each ICM generic, which was assessed for the overall severity and for each severity category. In addition, the incidence of ICM-related ADR according to premedication was also evaluated. To compare the incidence of ICM-related ADR among ICM generics, chi-square tests and multiple comparisons using chi-square test were performed. *P* < .05 was considered statistically significant, with Bonferroni correction adopted for multiple comparisons.^[16]^

#### 2.4.2. Literature search and study selection

A systematic review and meta-analysis were performed in accordance with the Preferred Reporting Items for Systematic Reviews and Meta-Analyses guidelines.^[17–19]^ The literature in PUBMED, EMBASE, and KOREAMED databases was systematically searched to identify original articles investigating the incidence of ICM-related ADR in the Republic of Korea, published up to December 31, 2021. The search terms were as follows: ((Korea)) AND ((“hypersensitivity reaction”) OR (“adverse drug reaction”)) AND ((“iodinated contrast media”) OR (“radiocontrast media”) OR (“CT contrast agent”)).

#### 2.4.3. Data extraction and quality assessment

The total numbers of contrast-enhanced CT examinations, the total number of CT examinations with ICM-related ADRs, the number of CT examinations with ICM-related ADRs for each generic profile of nonionic low-osmolar ICM (iobitridol, iohexol, iomeprol, iopamidol, iopromide, and ioversol), and the number of CT examinations with ICM-related ADRs for each severity category were collected from the eligible articles. The Risk of Bias for Nonrandomized Studies tool was used to assess study quality. The study selection, data extraction, and quality assessment were conducted by 2 reviewers independently.

#### 2.4.4. Systematic review and meta-analysis

Data extracted from the registered published literatures were integrated into data obtained from our institution and evaluated together. The pooled incidences of ICM-related ADR were obtained for the whole and for each ICM generic. The pooled incidences of ICM-related ADR for each severity category were also obtained. We calculated the pooled incidence using mixed-effects logistic regression models (binomial-normal model), instead of inverse-variance weighting models, because we investigated rare events.^[20]^ Heterogeneity was evaluated using Cochran Q-test and Higgins’ inconsistency index, with *P* < .1 and I^2^ > 50% indicating significant heterogeneity, respectively.^[21]^ All statistical analyses were performed using R version 4.03 (R foundation for Statistical Computing, Vienna, Austria), and MedCalc version 20.014 (MedCalc Software, Ostend, Belgium).

## 3. Results

### 3.1. Overall incidence and severity of ICM-related ADRs in Asan medical center

Among 1,184,935 contrast-enhanced CT examinations performed in Asan Medical Center, 23,516 CT examinations (1.98%) using iobitridol, iomeprol, and iopromide were excluded. Finally, a total of 1,161,419 CT examinations using iohexol, iopamidol, and ioversol were included (mean age ± standard deviation, 59.6 ± 12.5 years; male:female = 55:45). The overall incidence of ICM-related ADR was 1.26% (14,671/1,161,419). The incidences of mild, moderate, and severe ADRs were 1.15% (13,395/1,161,419; 91.3% of ADRs), 0.10% (1,128/1,161,419; 7.7% of ADRs), and 0.013% (148/1,161,419; 1.01% of ADRs), respectively. Of the 1,161,419 examinations, 106,337 were performed after premedication (9.2%) due to a previous history of ADR. The incidence of ICM-related ADR was 5.81% (6,178/106,337) in patients requiring premedication and 0.85% (9,019/1,058,412) in patients not requiring premedication.

When duplication was allowed, the most frequent signs and symptoms of ADR, with a frequency of ≥1% were as follows, in order: limited urticaria/pruritis (59.3%), nausea/vomiting (10.6%), dizziness (5.6%), nasal congestion/sneezing/rhinorrhea (4.6%), flushing (4.5%), diffuse urticaria/pruritis (3.6%), and facial edema (3.0%), cough (1.3%), hypotension (1.3%), dyspnea (1.2%), and headache (1.0%).

### 3.2. Incidence and severity of ICM-related ADRs according to ICM generics in Asan Medical Center

The incidence and severity of ICM-related ADRs according to ICM generic in Asan Medical Center are summarized in Table 1. Ioversol (1.86%, 3,539/190,467) resulted in the highest incidence of ADRs, followed by iopamidol (1.34%, 5,815/433,094) and iohexol (0.99%, 5,317/537,858). Even when the ICM-related ADRs of each generic were separately analyzed into a group requiring premedication and a group not requiring premedication, the ADR incidence according to the ICM generics showed a similar trend, and there was a significant difference among the ICM generics (*P* < .001) (Supplementary Table 1, http://links.lww.com/MD/I940).

**Table 1:** The incidence and severity of iodine contrast medium-related adverse drug reactions according to the generic profile of iodine contrast medium (present study).

ADR according to the generic profile of ICM					
Generic	ICM usage	Overall ADR (%)	Mild ADR (%)	Moderate ADR (%)	Severe ADR (%)
Iohexol	537,858	5,317 (0.99)	4,864 (0.90)	393 (0.07)	60 (0.011)
Iopamidol	433,094	5,815 (1.34)	5,340 (1.23)	435 (0.10)	40 (0.009)
Ioversol	190,467	3,539 (1.86)	3,191 (1.68)	300 (0.16)	48 (0.025)
Total	1,161,419	14,671 (1.26)	13,395 (1.15)	1,128 (0.10)	148 (0.013)
Pairwise comparisons of ADR using Bonferroni correction					
Generic		Overall ADR	Mild ADR	Moderate ADR	Severe ADR
Iohexol vs Iopamidol		*P* < .001	*P* < .001	*P* < .001	*P* = .354
Iopamidol vs Ioversol		*P* < .001	*P* < .001	*P* < .001	*P* < .001
Iohexol vs Ioversol		*P* < .001	*P* < .001	*P* < .001	*P* < .001

Data are the number of patients, with percentages in parentheses.

ADR = adverse drug reaction, ICM = iodinated contrast media

Each incidence of mild and moderate ADR exhibited a similar trend to the overall incidence (Supplementary Table 2, http://links.lww.com/MD/I941). For severe ADR, ioversol (0.025%, 48/190,367) had the highest incidence, followed by iohexol (0.011%, 60/537,858) and iopamidol (0.009%, 40/433,094). Pairwise comparisons of iohexol, iopamidol, and ioversol revealed that the ADR incidences were statistically significantly different for overall severity and for each severity category (*P* < .001), except that iohexol and iopamidol did not differ in severe ADR (*P* = .354).

### 3.3. Pooled incidence and severity of ICM-related ADRs in the Republic of Korea

**Figure 1.**
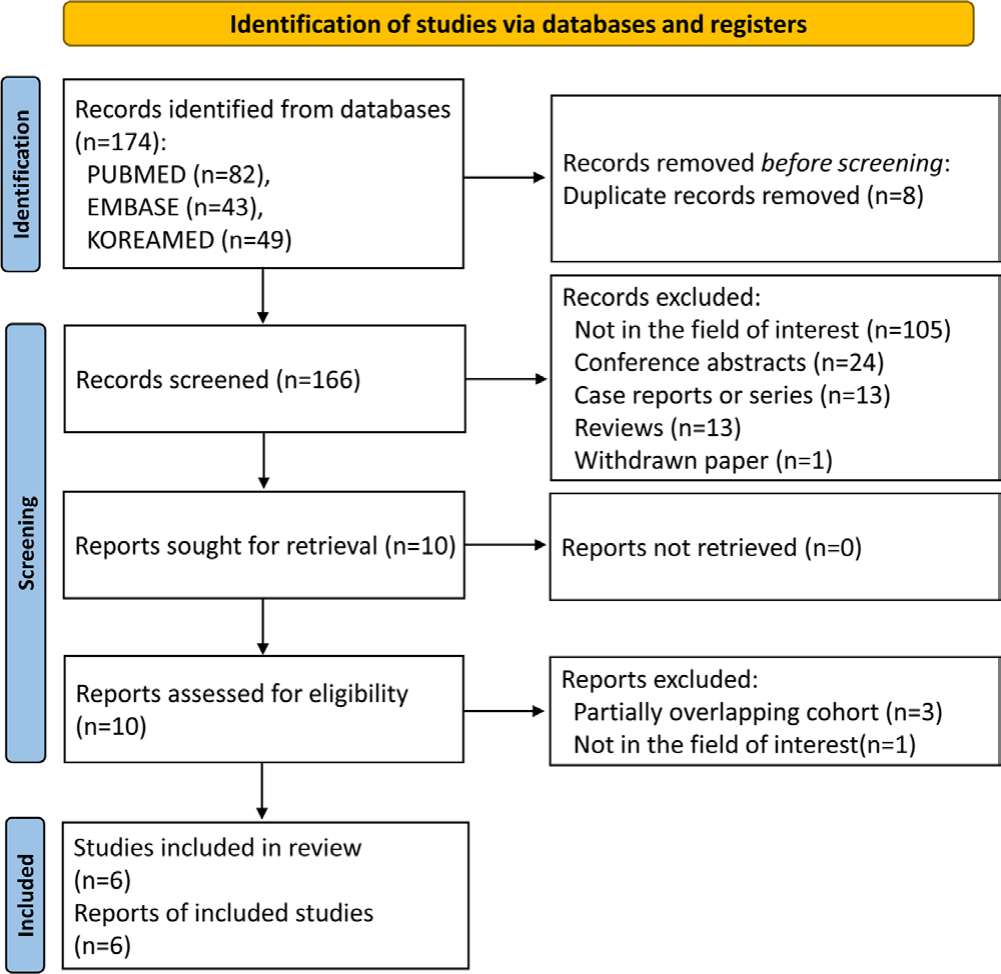
Flow diagram of the study selection process.

**Figure 2.**
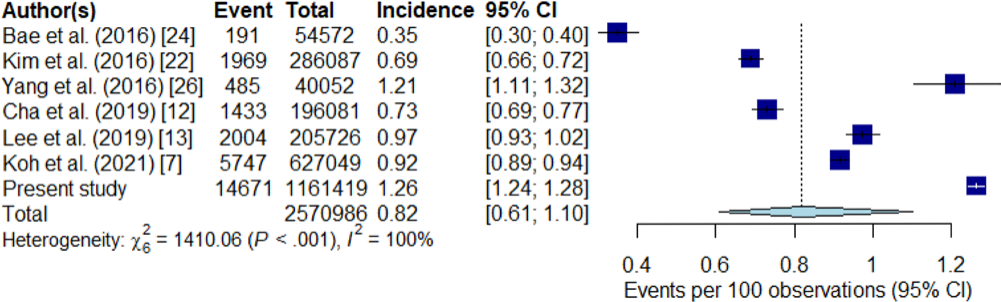
Forest plot of the overall pooled incidence of iodine contrast medium-related adverse drug reactions. Numbers are pooled estimated with 95% confidence intervals (CIs) in parentheses; horizontal lines indicate 95% CIs.

Seven studies (including our study) investigating the incidence of ICM-related ADR in the Republic of Korea, involving 2,570,986 contrast-enhanced CT examinations from 12 institutions, were finally included in the meta-analysis (Fig. 1, Table 2). All 7 studies were regarded as having an unclear risk of bias in the participant comparability domain and high risk of bias in the confounding variables domain (Supplementary Fig. 1, http://links.lww.com/MD/I942). The overall pooled incidence of ICM-related ADRs was 0.82% (95% CI: 0.61%–1.10%) (Fig. 2). The pooled incidences of mild, moderate, and severe ADRs were 0.72% (95% CI: 0.50%–1.04%), 0.11% (95% CI: 0.08%–0.15%), and 0.013% (95% CI: 0.010%–0.018%), respectively (Table 3). Inter-study heterogeneity was observed for the overall ADR incidence (*P* < .001, I^2^ = 100%) and for each severity category (mild, *P* < .001, I^2^ = 100%; moderate, *P* < .001, I^2^ = 98%; severe, *P* = .001, I^2^ = 75%).

**Table 2:** Characteristics of the selected studies.

Author (yr of publication)	Institution	Duration of patient recruitment	No. of examinations	No. of generic profile of ICM	Study design	Consecutive enrollment
Bae et al (2016)^[22]^	Gyeongsang National University Changwon Hospital	2010.06–2013.05	54,572	4 (iodixanol, iohexol, iomeprol, iopromide)	Prospective	Yes
Kim et al (2016)^[23]^	Severance Hospital	2006.01–2010.12	286,087	4 (iobitridol, iohexol, iopamidol, iopromide)	Retrospective	Yes
Yang et al (2016)^[24]^	Seoul National University Bundang Hospital	2011.06–2012.05	40,052	4 (iodixanol, iohexol, iomeprol, iopromide)	Retrospective	Yes
Cha et al (2019)^[12]^	Seven tertiary referral hospitals*	2017.03–2017.10	196,081	6 (iobitridol, iohexol, iomeprol, iopamidol, iopromide, ioversol)	Retrospective	Yes
Lee et al (2019)^[13]^	Seoul National University Hospital	2012.07–2014.06	205,726	5 (iobitridol, iohexol, iomeprol, iopamidol, iopromide)	Retrospective	Yes
Koh et al (2021)^[7]^	Samsung Medical Center	2015.01–2018.12	627,049	6 (iobitridol, iohexol, iomeprol, iopamidol, iopromide, ioversol)	Retrospective	Yes
Present study†	Asan Medical Center	2016.01–2021.12	1,161,419	3 (iohexol, iopamidol, ioversol)	Retrospective	Yes

* Seoul National University Hospital, Kyungpook National University Hospital, Chungbuk National University Hospital, Pusan National University Yangsan Hospital, Chonnam National University Hospital, Gyeongsang National University Hospital, and Chung-Ang University Hospital

† The data of the present study were also included in the analysis.

ICM = iodinated contrast media.

**Table 3:** The overall incidence and severity of iodine contrast medium-related adverse drug reactions.

	ICM usage	ADR (%)	Mild ADR (%)	Moderate ADR (%)	Severe ADR (%)
Bae et al (2016)^[22]^	54,572	191 (0.35)	157 (0.29)	29 (0.05)	5 (0.009)
Kim et al (2016)^[23]^	286,087	1,969 (0.69)	NA	NA	NA
Yang et al (2016)^[24]^	40,052	485 (1.21)	440 (1.10)	54 (0.13)	9 (0.022)
Cha et al (2019)^[12]^	196,081	1,433 (0.73)	1,192 (0.61)	224 (0.11)	17 (0.009)
Lee et al (2019)^[13]^	205,726	2,004 (0.97)	1,748 (0.85)	210 (0.10)	46 (0.022)
Koh et al (2021)^[7]^	627,049	5,747 (0.92)	4,502 (0.72)	1,173 (0.19)	72 (0.011)
Present study	1,161,419	14,671 (1.26)	13,395 (1.15)	1,128 (0.10)	148 (0.013)
Pooled incidence (95% CI)	2,570,986	0.82 (0.61–1.10)	0.72 (0.50–1.04)	0.11 (0.08–0.15)	0.013 (0.010–0.018)

Data are the number of patients, with percentages in parentheses.

ADR = adverse drug reaction, ICM = iodinated contrast media.

**Figure 3.**
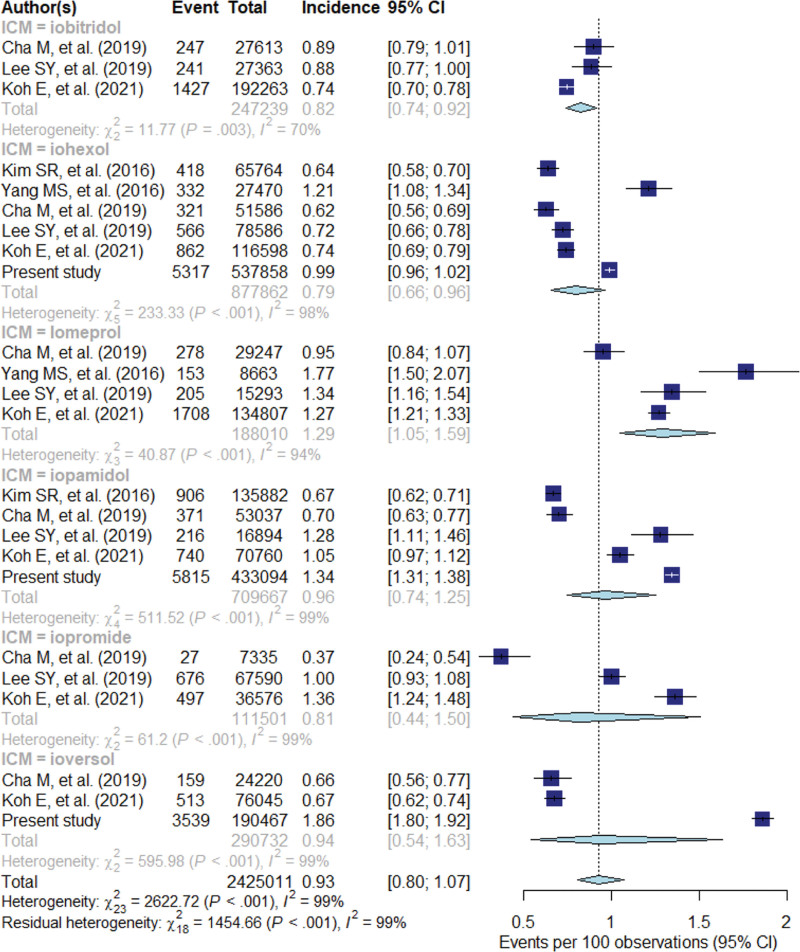
Forest plot of pooled incidences of iodine contrast medium (ICM)-related adverse drug reactions according to the generic profile of ICM. Iobitridol (A), iohexol (B), iomeprol (C), iopamidol (D), iopromide (E), ioversol (F). Numbers are pooled estimated with 95% confidence intervals (CIs) in parentheses; horizontal lines indicate 95% CIs.

**Figure 4.**
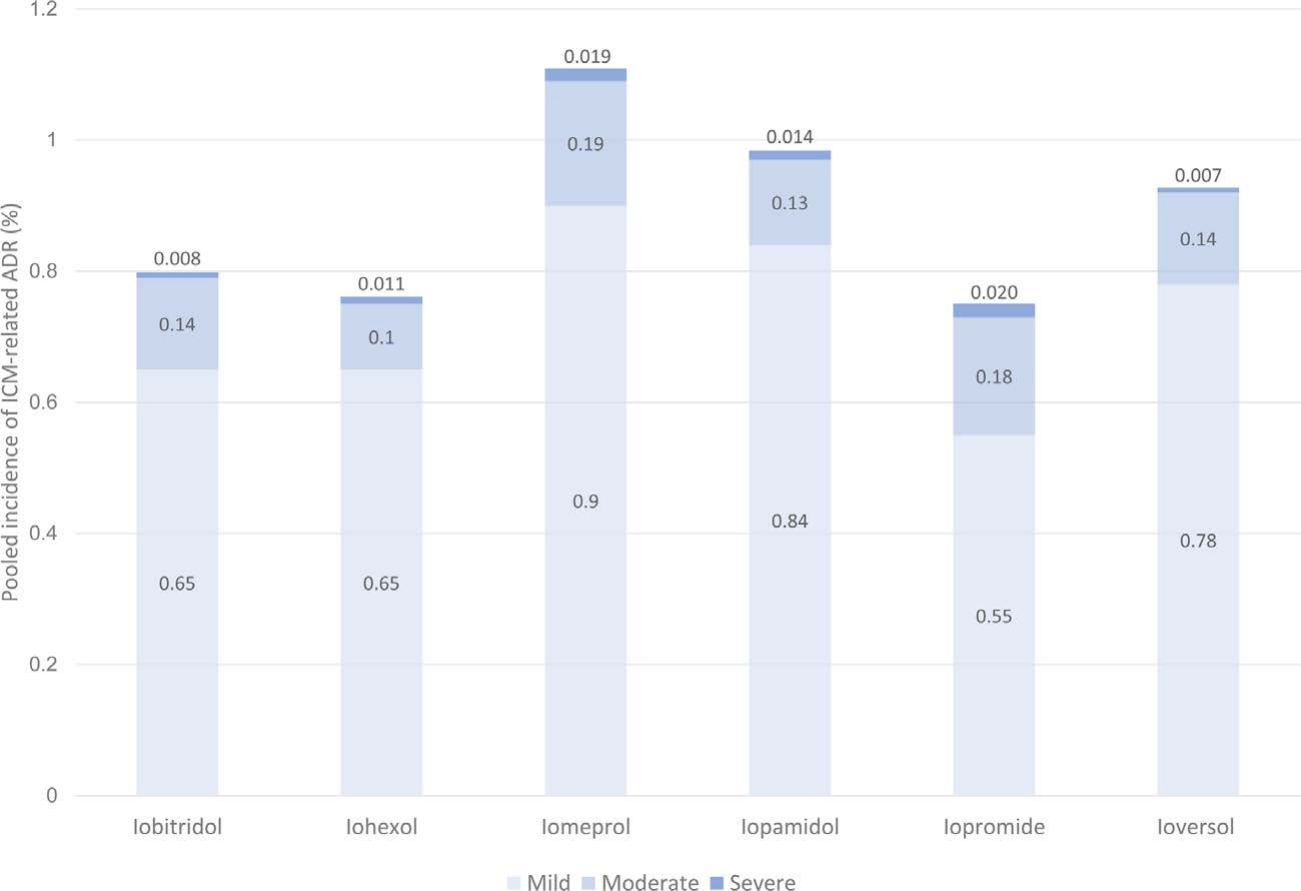
The pooled incidence and severity of iodine contrast medium (ICM)-related adverse drug reactions according to the generic profile of ICM.

The pooled incidences of ADRs according to ICM generics are shown in Figure 3. Iomeprol (1.29%, 95% CI: 1.05%–1.59%) had the highest pooled incidence of ADRs, followed by iopamidol (0.96%, 95% CI: 0.74%–1.25%), ioversol (0.94%, 95% CI: 0.54%–1.63%), iobitridol (0.82%, 95% CI: 0.74%–0.92%), iopromide (0.81%, 95% CI: 0.44%–1.50%), and iohexol (0.79%, 95% CI: 0.66%–0.96%). Inter-study heterogeneity was noted for all ICM generics, and significant residual heterogeneity was present (*P* < .001, I^2^ = 99%). In multiple pairwise comparisons, in most cases, there were no significant differences in the pooled incidences of ADRs between ICMs, except between iomeprol and iobitridol (*P *< .001) and between iomeprol and iohexol (*P < *.001). The pooled incidences of ADRs for each severity category according to ICM generics were also evaluated (Fig. 4, Supplementary Table 2, http://links.lww.com/MD/I941). For each severity category, there were no statistically significant differences between ICMs, except for mild ADR between iomeprol and iobitidol (*P* < .001).

## 4. Discussion

We reported the incidence and severity of ICM-related ADRs for 6 generic nonionic low-osmolar ICMs in the Republic of Korea, adding data from Asan Medical Center. In our single-center study, the overall incidence of ICM-related ADR was 1.26%. The incidences of mild, moderate, and severe ADRs were 1.15%, 0.10%, and 0.013%, respectively. The pooled incidences of overall, mild, moderate, and severe ICM-related ADRs in the Republic of Korea were 0.82%, 0.72%, 0.11%, and 0.013%, respectively. Multiple pairwise comparisons showed no significant differences in the overall pooled incidence of ICM-related ADRs between ICM generics, except iomeprol versus iobitridol (1.29% vs 0.82%) and iomeprol vs iohexol (1.29% vs 0.79%). For moderate and severe ADRs, there were no significant differences between ICMs.

A recent study identified that frequencies of certain HLA alleles, including DRB1*15:02, were significantly higher in patients with ICM-related anaphylaxis in the Korean population.^[14]^ A family history of ICM-related hypersensitivity reaction was also shown to predict occurrence of such a reaction, suggesting a potential genetic predisposition.^[12]^ Therefore, as a single ethnic group, we considered it necessary to report the incidence and severity of ICM-related ADRs using only domestic data from the Republic of Korea. Previous studies on domestic data have reported the incidence of ICM-related ADRs according to ICM generics, but yielded inconsistent results: Koh et al reported that iopromide (1.36%) had the highest ADR incidence, followed by iomeprol (1.27%), iopamidol (1.05%), iobitridol (0.74%), iohexol (0.74%), and ioversol (0.67%), with significant differences among ICMs in overall severity and in each severity category;^[7]^ Kim et al reported that the immediate ADR incidence was significantly higher with iopromide (1.03%) and lower with iobitridol (0.34%);^[23]^ Cha et al found that iomeprol (0.95%) and iobitridol (0.89%) resulted in significantly higher incidences of hypersensitivity reactions, whereas iohexol (0.62%) and iopromide (0.37%) were associated with significantly lower incidences of hypersensitivity reactions;^[12]^ and, Lee et al found no significant difference in the overall immediate hypersensitivity reaction incidences among ICMs.^[13]^ This inconsistency is thought to be in part to the relatively small study population; therefore, we performed further investigations with a larger population and meta-analysis. To the best of our knowledge, no previous meta-analysis on the incidence and severity of ICM-related ADRs in the Republic of Korea has been reported. The European Society of Urogenital Radiology guidelines on contrast media state that there is no difference in the incidence of ADRs among the nonionic low-osmolar agents;^[25]^ however, the Korean guidelines on contrast media do not have a clear guideline for selecting ICM generics.^[8]^ Knowing whether there is a difference in the incidence of ADRs among the ICMs can be clinically useful as it allows for providing guidance on ICM selection.

Our results suggest that there is no significant difference in the incidence of ICM-related ADRs, particularly moderate-to-severe ADRs, among the nonionic low-osmolar ICMs, that is, major problems that require medical management and can lead to life-threatening conditions. These results are also consistent with those of a previous meta-analysis.^[10]^ That study included data up to 2017, from both the East and the West, and excluded all papers that did not investigate the ADR incidence of at least one ICM generic type and those not published in English. Of the studies included in our meta-analysis, only 2 studies (Kim et al and Yang et al) were included in the previous meta-analysis. Although their overall pooled incidence of ICM-related ADRs was higher than in our study, at 1.03%, the pooled incidence of severe ADRs was 0.0141%, which was similar to our result. The reason for the lower overall pooled incidence in our study may be due to genetic differences or a decrease in the number of mild ADRs, including more recent data after systematizing the management of ICM-related ADR.^[22]^ In the previous meta-analysis, iomeprol had the highest overall pooled incidence, but no significant differences were found between ICM generics, in line with our results.^[10]^

Substantial inter-study heterogeneity was observed in our meta-analysis. A variety of the factors could be considered. The characteristics of the inclusion criteria—inclusion or exclusion of delayed reactions and immediate reactions, physiologic and allergic-like reactions, and outpatients only or both outpatients and inpatients—varied across studies.^[7,13,22,23,26]^ Although not clearly stated in each study, there may be differences in the premedication protocol among the institutions; also, the proportion of high-risk and pre-medicated patients may have been different. Because systematic reporting and management protocols for ICM-related ADRs were not previously well established, the results may also have been affected by differences in the timing of each study.^[22,26]^ The preference and total usage of each ICM by an institution may have varied depending on individual circumstances, such as competitive bidding.

There were several limitations to our study. First, the injection rate, total dose, and iodine concentration of each contrast agent were not considered. Although no significant relationship was found between the dose or iodine concentration of the contrast agent and the incidence of ICM-related ADRs in previous studies,^[7,12,24,27]^ further detailed analysis is required. Second, various potential positive or negative risk factors (e.g., individual history of ICM usage and ICM-related ADR, with or without premedication or replacement of contrast media) could not be considered. However, we attempted to minimize this limitation by including a large number of patients (>1,000,000 in our institution alone) to evaluate the incidence of ICM-related ADRs according to ICM generics. A recent study demonstrated that switching ICM generics based on the presence of a common N-(2,3-dihydroxypropyl) carbamoyl side-chain helped to reduce the recurrence of severe ICM-related hypersensitivity reaction,^[28]^ and further study is needed. Third, our institutional data for iobitridol, iomeprol, and iopromide were excluded from the meta-analysis to ensure unbiased measures. The incidence of the other 3 ICMs could have been over- or under-estimated because our institution had the largest number of patients among the included studies.

In conclusion, there were no significant differences in the overall pooled incidences of ICM-related ADRs between nonionic low-osmolar ICM generics, except for iomeprol versus iobitridol/iohexol. Of note, there were no significant differences between ICM generics in the pooled incidences of moderate and severe ICM-related ADRs. Our results suggest that no restriction is required for selection among nonionic low-osmolar ICMs.

## Author contributions

**Conceptualization:** Eun Bee Jang, Chong Hyun Suh, Choong Wook Lee.

**Data curation:** Eun Bee Jang, Chong Hyun Suh.

**Formal analysis:** Eun Bee Jang, Chong Hyun Suh, Pyeong Hwa Kim.

**Funding acquisition:** Chong Hyun Suh, Choong Wook Lee.

**Investigation:** Eun Bee Jang, Chong Hyun Suh.

**Methodology:** Chong Hyun Suh.

**Project administration:** Chong Hyun Suh, Choong Wook Lee.

**Resources:** Chong Hyun Suh, Choong Wook Lee.

**Software:** Chong Hyun Suh.

**Supervision:** Chong Hyun Suh, Ah Young Kim, Kyung-Hyun Do, Jeong Hyun Lee, Ah Young Jung, Choong Wook Lee.

**Validation:** Chong Hyun Suh, Dong-Il Gwon.

**Visualization:** Chong Hyun Suh.

**Writing – original draft:** Eun Bee Jang, Ah Young Jung.

**Writing – review & editing:** Chong Hyun Suh, Pyeong Hwa Kim, Ah Young Kim, Kyung-Hyun Do, Jeong Hyun Lee, Dong-Il Gwon, Choong Wook Lee.

## Supplementary Material






