# Supplementary material for: Incidence and severity of nonionic low-osmolar iodinated contrast medium-related adverse drug reactions in the Republic of Korea: Comparison by generic
Source: Medicine (Baltimore). 2023 May 12;102(19):e33717. doi: 10.1097/MD.0000000000033717 (PMC10174392; doi:10.1097/MD.0000000000033717)
Supplement: Supplementary file 1 [file medi-102-e33717-s001.pdf]

Supplementary Table 1. The Incidence of Iodine Contrast Medium-related Adverse Drug Reactions, according to whether Premedication was used (Present study)

**Generic**	**Premedication (+)**		**Premedication (-)**	
	**Number**	**ADR (%)**	**Number**	**ADR (%)**
Iohexol	40,631	2139 (5.26)	497,227	3178 (0.64)
Iopamidol	36,689	1981 (5.40)	396,405	3834 (0.98)
Ioversol	25,687	1532 (5.96)	164,780	2007 (1.22)
Total	106,337	6178 (5.81)	1,058,412	9019 (0.85)

Data are the number of patients, with percentages in parentheses.

ADR = adverse drug reaction
