# Supplementary material for: Incidence and severity of nonionic low-osmolar iodinated contrast medium-related adverse drug reactions in the Republic of Korea: Comparison by generic
Source: Medicine (Baltimore). 2023 May 12;102(19):e33717. doi: 10.1097/MD.0000000000033717 (PMC10174392; doi:10.1097/MD.0000000000033717)
Supplement: Supplementary file 2 [file medi-102-e33717-s002.pdf]

Supplementary Table 2. The Incidence and Severity of Iodine Contrast Medium-Related Adverse Drug Reactions according to the Generic Profile of Iodine Contrast Medium.

**Generic**	**Severity**	**Cha et al. (2019) [12]**	**Koh et al. (2021) [7]**	**Present study**	**Pooled incidence (95% CI)**
Iobitridol	Mild (%)	203 (0.74)	1156 (0.60)	NA	0.65% (0.57–0.75)
	Moderate (%)	42 (0.15)	255 (0.13)	NA	0.14% (0.12–0.15)
	Severe (%)	2 (0.007)	16 (0.008)	NA	0.008% (0.005–0.013)
	ADR (%)	247 (0.89)	1427 (0.74)	NA	
	ICM usage	27,613	192,263	NA	
Iohexol	Mild (%)	270 (0.52)	660 (0.57)	4864 (0.90)	0.65% (0.49–0.85)
	Moderate (%)	46 (0.09)	192 (0.16)	393 (0.07)	0.10% (0.07–0.15)
	Severe (%)	6 (0.012)	10 (0.009)	60 (0.011)	0.011% (0.009–0.013)
	ADR (%)	321 (0.62)	862 (0.74)	5317 (0.99)	
	ICM usage	51,586	116,598	537,858	
Iomeprol	Mild (%)	238 (0.81)	1302 (0.97)	NA	0.90% (0.80–1.02)
	Moderate (%)	36 (0.12)	379 (0.28)	NA	0.19% (0.11–0.34)
	Severe (%)	4 (0.014)	27 (0.020)	NA	0.019% (0.013–0.027)
	ADR (%)	278 (0.95)	1708 (1.27)	NA	
	ICM usage	29,247	134,807	NA	
Iopamidol	Mild (%)	320 (0.60)	578 (0.82)	5340 (1.23)	0.84% (0.61–1.19)
	Moderate (%)	47 (0.09)	156 (0.22)	435 (0.10)	0.13% (0.08–0.20)
	Severe (%)	4 (0.008)	27 (0.038)	40 (0.009)	0.014% (0.006–0.033)
	ADR (%)	371 (0.70)	740 (1.05)	5815 (1.34)	
	ICM usage	53,037	70,760	433,094	
Iopromide	Mild (%)	20 (0.27)	390 (1.07)	NA	0.55% (0.21–1.43)
	Moderate (%)	7 (0.10)	98 (0.27)	NA	0.18% (0.08–0.36)
	Severe (%)	0	9 (0.025)	NA	0.020% (0.011–0.039)
	ADR (%)	27 (0.37)	497 (1.36)	NA	
	ICM usage	7335	36,576	NA	
Ioversol	Mild (%)	124 (0.51)	416 (0.55)	3191 (1.68)	0.78% (0.42–1.45)
	Moderate (%)	35 (0.14)	93 (0.12)	300 (0.16)	0.14% (0.12–0.17)
	Severe (%)	0	4 (0.005)	48 (0.025)	0.007% (0.001–0.033)
	ADR (%)	159 (0.65)	513 (0.67)	3539 (1.86)	
	ICM usage	24,220	76,045	190,467	

Data are the number of patients, with percentages in parentheses.

ICM = iodinated contrast media, ADR = adverse drug reaction
