# Supplementary material for: Incidence and severity of nonionic low-osmolar iodinated contrast medium-related adverse drug reactions in the Republic of Korea: Comparison by generic
Source: Medicine (Baltimore). 2023 May 12;102(19):e33717. doi: 10.1097/MD.0000000000033717 (PMC10174392; doi:10.1097/MD.0000000000033717)

Supplementary Figure 1. Study quality assessment of the 7 studies that reported the incidence of iodine contrast medium-related adverse drug reactions in the Republic of Korea. ROBANS = Risk of Bias for Nonrandomized Studies

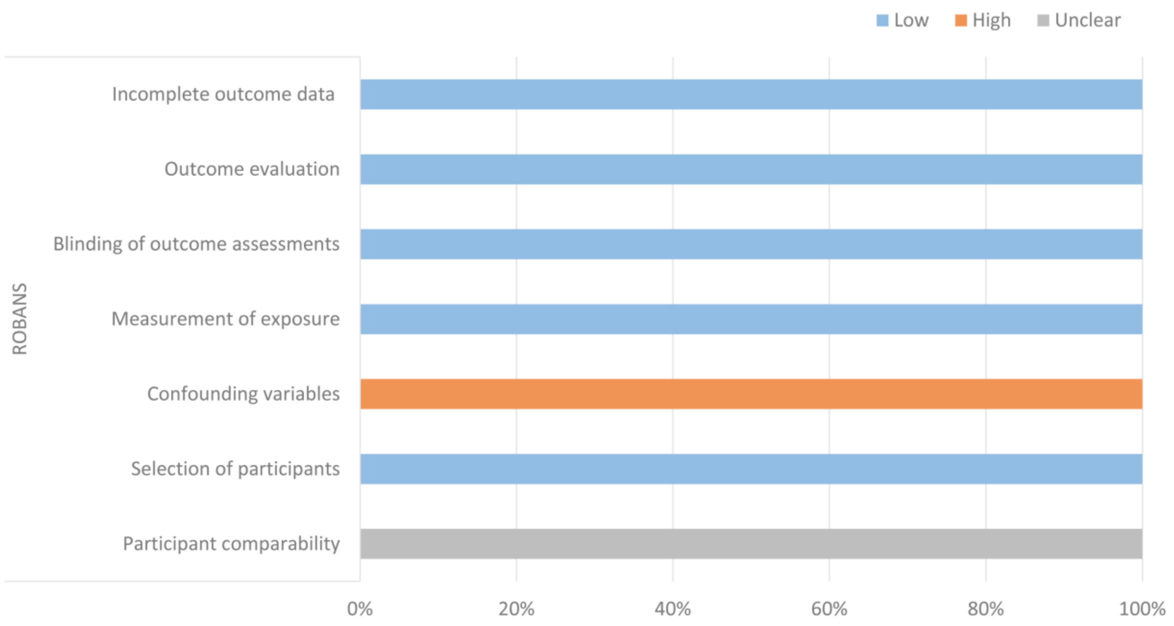

Supplement: Supplementary file 3 [file medi-102-e33717-s003.pdf]
